# Technical Efficiency of Prevention Services for Functional Dependency in Japan’s Public Long-term Care Insurance System: An Ecological Study

**DOI:** 10.34172/ijhpm.8226

**Published:** 2024-06-19

**Authors:** Ayumi Hashimoto, Hideki Hashimoto, Hiroyuki Kawaguchi

**Affiliations:** ^1^Graduate School of Medicine, The University of Tokyo, Tokyo, Japan.; ^2^Department of Health and Social Behavior, School of Public Health, The University of Tokyo, Tokyo, Japan.; ^3^Economics Faculty, Seijo University, Tokyo, Japan.

**Keywords:** Universal Health Coverage, Long-term Care Insurance, Preventive Benefits, Efficiency, Japan

## Abstract

**Background:**

Public long-term care insurance (LTCI) systems can promote equal and wider access to quality long-term care. However, ensuring the financial sustainability is challenging owing to growing care demand related to population aging. To control growing demand, Japan’s public LTCI system uniquely provided home- and community-based prevention services for functional dependency for older people (ie, adult day care, nursing care, home care, functional screening, functional training, health education, and support for social activities), following nationwide protocols with decentralized delivery from 2006 until 2015. However, evaluations of the effects of these services have been inconclusive.

**Methods:**

We estimated the marginal gain and technical efficiency of local prevention services using 2009–2014 panel data for 474 local public insurers in Japan, based on stochastic frontier analysis. The outcome was the transformed sex-and age-adjusted ratio of the observed to expected number of individuals aged ≥65 years certified for moderate care. Higher outcome values indicate lower population risk of moderate functional dependency in each region in each year. The marginal gains of the provided quantities of prevention services as explanatory variables were estimated, adjusting for regional medical and welfare access, care demand and supply, and other regional factors as covariates.

**Results:**

Prevention services (except functional screening) significantly reduced the population risk of moderate functional dependency. Specifically, the mean changes in outcome per 1% increase in adult day care, other nursing care, and home care were 0.13%, 0.07%, and 0.04%, respectively. The median technical efficiency of local public insurers was 0.94 (interquartile range: 0.89–0.99).

**Conclusion:**

These findings suggest that population-based services with decentralized local operation following standardized protocols could achieve efficient prevention across regions. This study could inform current discussions about the range of benefit coverage in public LTCI systems by presenting a useful option for the provision of preventive benefits.

## Background

**Key Messages**

**Implications for policy makers**

- Japan’s public long-term care insurance (LTCI) system provides prevention services.
- Local public insurers operate highly efficient services across regions.
- Preventive benefits could reduce the population risk of functional dependency.
- Decentralized operation with standardized control may be a feasible policy scheme.

**Implications for the public**

Public long-term care insurance (LTCI) systems can help to provide equal and wider access to high quality long-term care. However, an important challenge is maintaining financial sustainability. In this study, we examined the unique introduction of home- and community-based prevention services for functional dependency for older people in Japan to address the increasing demand for care by the aging population. These services are provided as part of universal health coverage and follow a national protocol with decentralized delivery. Our findings indicate that public LTCI insurers in Japan operate highly efficient prevention services. Decentralized operation and standardized quality control may achieve equivalent service efficiency across regions. The present findings contribute to existing discussions about the range of benefit coverage in public LTCI systems by presenting a useful option for the provision of preventive benefits.

The introduction of public long-term care insurance (LTCI) has become a key theme in discussions of policies for the aging population.^1,2^ Although the introduction of LTCI systems likely ensures equal and wider access to quality long-term care, an important challenge is maintaining financial sustainability in the context of increasing demand for care. Recent discussions have mainly focused on the financing schemes and structure of benefit coverage to balance demand and supply in the long term. In this context, the policy of focusing benefit coverage on individuals with the highest care needs has been prioritized in many countries to ensure efficient resource allocation given limited financial resources.^3,4^ However, the optimal allocation of resources among people with mild care needs is an ongoing debate.^2,3^

 In 2000, Japan introduced a public LTCI system that covers older people aged ≥65 years with a wide range of care needs.^1,5-7^ Shortly after its introduction, the system experienced rapid increases in demand and expenditure, which the Japanese government addressed through service payment reductions for providers and additional charges on accommodation and meal fees for nursing home users. In 2006, a unique policy was introduced for individuals requiring mild care, instead of limiting benefits to those with greater needs. In an attempt to control the long-term rise in care demand, the government introduced a new eligibility criteria category for users with mild care needs to provide preventive benefits (called Yobo-Kyufu).

 Japan’s LTCI system uses a multi-payer system that comprises municipality local governments or allied institutions.^5^ A national standardized protocol for home- and community-based preventive benefits was universally applied to local service implementation for older people who require assistance in daily life.^1,6,7^ Preventive benefits were provided to 3.8% of the population aged ≥65 years as of 2006.^8^ In addition, local LTCI insurers were mandated to provide community programs on prevention for functional dependency that permitted local discretion according to local needs.^9,10^ Since 2015, a proportion of preventive benefits was transferred to independent local welfare programs that were no longer covered by LTCI benefits.^1,8,9^

 The expectation was that preventive services would potentially control the demands and expenditure of long-term care,^11^ though the effectiveness and efficiency of public policy practices have not been systematically demonstrated. Several studies conducted in Japan have reported mixed results for the effectiveness of preventive benefits and community programs in the LTCI system.^12-16^ Because these studies were conducted in a small number of local municipalities, the mixed findings could be explained by performance differences in decentralized operations and regional characteristics. Another reason for the inconsistent findings may be that these studies relied on individual-level outcomes that were not fully adjusted for individual health/comorbidity conditions.

 To overcome these challenges, we estimated the marginal gains of prevention services for functional dependency, taking into account performance differences across local regions and using national data from LTCI insurers. Instead of using individual-level data, we used an ecological design, taking each LTCI insurer as a unit of analysis. This allowed associations between prevention services and functional dependency to be evaluated separately from performance disparities among LTCI insurers.

## Methods

###  Local Prevention Services in Japan’s LTCI

 People aged ≥65 years and those aged 40–64 years with aging-related disease are eligible for LTCI benefits. Eligibility is assessed solely based on care needs through a nationally standardized process.^5,9^ There are seven eligibility levels: the two lowest levels are “assistance required levels” (AL) 1–2, and the remaining five levels are “care required levels” (CL) 1–5. Each level has a benefit ceiling amount, and certified older people pay a 10%–30% co-payment according to income level.

 LTCI insurers provide preventive benefits for individuals certified for AL1–2.^5,7-9^ The target populations of community programs vary by LTCI insurers and programs, and include those not certified for LTCI benefits (eg, functional screening and functional training) and community-dwelling older people (eg, health education and social activities).^5,7-9^ Preventive benefits consist of adult day care (eg, nursing care, rehabilitation, and recreation in adult day care centers), home care (eg, personal care, housekeeping, bathing, nurse visits, rehabilitation, and medical management), and other nursing care (eg, care management, care home with daily living support, home remodeling, assistive devices, short-stay respite care, dementia group homes, and mixed and flexible care). Community programs (which are mostly free of charge) include functional screening using the validated Kihon Checklist questionnaire, functional training (eg, physical, cognitive, oral function training, and nutritional intervention), health education (eg, lectures, consultations, and classes), and support for social activities (eg, exercise, recreation, and volunteering).^10,12^

 The central government has implemented several measures to control the quality of local services.^5,7,9^ Preventive benefit providers are licensed and supervised by prefectural governments, and are required to adhere to the national protocol.^17^ There are specifications for structural requirements, including personnel (eg, number of staff and qualifications) and facilities (eg, type and size of rooms). There are standardized protocols for delivery (eg, assessment, care plans, and care coordination) and management (eg, work schedules, emergency responses, hygiene, privacy protection, and accident and abuse prevention). To better meet local needs, municipalities are permitted to use discretion in setting the community program target populations, delivery mode, contents, and frequency.^9,10^

###  Sample and Data Sources

 Using public government reports on LTCI, demographics, and living environment,^8,10,18-21^ we compiled panel data for LTCI insurers from 2009 to 2014, a period characterized by a stable supply of preventive benefits and community programs with no major changes in the LTCI act, and for which local insurer unit data were available.^9,10^ First, a list of all 1741 municipalities in Japan was prepared according to the situation in October 2014, taking into account municipal mergers and incorporations. We excluded 222 municipalities affected by the 2011 Great East Japan Earthquake^22^ to remove the effects of the earthquake on living environment. The remaining 1519 municipalities operate 1366 LTCI insurers, which was our unit of analysis (some small municipalities jointly operate LTCI insurers). To reduce random variations in outcomes and explanatory variables owing to smaller population size and to maintain sample homogeneity, we limited to 476 insurers with populations of 50 000 to <2 000 000 as of 2009. From the total data (n = 2856; 476 insurers over 6 years), insurer data with missing observations in a year (n = 45; health education and support for social activities) were excluded for that year, and insurer data that contained outliers of unrealistically high numbers in any data were excluded for all years (n = 12; support for social activities). The final sample included 474 insurers (n = 2799), which comprised unbalanced panel data (433 insurers over 6 years, 37 insurers over 5 years, and 4 insurers over 4 years). The flowchart of sample selection is shown in Figure S1 in Supplementary file 1.

###  Outcome, Explanatory Variables, and Covariate Factors

 The ultimate goal of prevention services in LTCI is to reduce the number of people who are functionally dependent and require long-term care.^9^ Functional dependency is defined as the inability to perform basic activities of daily living (BADL) and instrumental activities of daily living (IADL) independently.^23^ The eligibility levels of AL 1–2, CL 1–2, and CL 3–5 reflect the level of care needed based on functional dependency, and represent mild, moderate, and severe care needs, respectively. Specifically, AL 1–2 beneficiaries are mostly independent in BADL such as bathing, toileting, and clothing, but require care in performing IADL such as going out and shopping. This category was the main target for prevention services in this study. CL 1–2 beneficiaries require partial support for BADL, such as bathing, and for IADL, such as cooking and managing money. CL 3–5 beneficiaries require full support for BADL, including toileting, clothing, and feeding. Individuals in CL 1–2 and 3–5 are eligible for formal social and institutional care rather than prevention services. Following the statistics for transition of care needs^24^ and a previous study,^15^ we assumed that effective preventive services for individuals certified as AL 1–2 status and the non-eligible subpopulation would reduce the likelihood of becoming CL 1–2 status. Most individuals certified for AL 1–2 did not change their functional dependency level in 1 year: 83% remained at AL 1–2, 15% shifted to CL 1–2, and only 2% shifted to CL 3–5 in 2014, respectively.^24^ One study in Japan found that preventive benefits avoid deteriorations from AL 1 to CL 1 or worse among individuals aged ≥85 years,^15^ suggesting that the main target to prevent deterioration is the shift from AL 1–2 to CL 1–2. Moreover, the prevalence of functional dependency is higher in women than in men and increases with age.^25^

 Based on the above points, the target outcome was the sex- and age-adjusted ratio of the observed to expected number of individuals aged ≥65 years certified as CL 1–2 with transformation (transformed O/E ratio); higher outcome values indicate a lower population risk of moderate functional dependency in a particular region in a particular year. The O/E ratio was adjusted for sex and age using the expected number of individuals for CL 1–2 by sex and age for each insurer. Specifically, the expected number of individuals was calculated by multiplying the national average sex- and 5-year age group-stratified certification rate for CL 1 and CL 2 by the corresponding sex- and 5-year age group-stratified population number for each insurer, and summing them.^8,19^ The O/E ratio is the observed number of individuals for CL 1–2 divided by the obtained expected number of individuals for CL 1–2. When calculating the transformed O/E ratio, we subtracted the O/E ratio from the median O/E ratio to reverse the positive and negative values for ease of interpretation, and zero values were replaced with 0.01 for log transformation using the Cobb–Douglas functional form.

 For the explanatory variables, we compiled quantities of all prevention services operated by LTCI insurers, namely, preventive benefits (ie, adult day care, home care, and other nursing care) and community programs (ie, functional screening, functional training, health education, and support for social activities). For 2011, data for the number of support for social activities conducted were not reported^10^; therefore, an average of these data for 2010 and 2012 was used for 2011.

 We also included covariate factors in the model for the reasons described below. To gauge access to medical care and social welfare resources, the number of general hospitals and clinics^21^ and social welfare costs for older people^20^ were used, respectively. For long-term care demand, the proportion of single households was used as an indicator of household informal care capacity.^19^ For long-term care supply, prefecture-level numbers of home- and community-based long-term care providers were used. This was because care providers are certified by the prefectural government and provide services in multiple municipalities within a prefecture; additionally, only prefecture-level data were available.^18^ For other regional factors, we used a financial capacity index (in which higher values reflect greater financial resources of the local government)^20^ and population density,^19^ which indicates the abundance and convenience of outdoor spaces and transportation. A year dummy was included to reflect changes in care demand and supply in response to a policy that restricted preventive benefits coverage from 2015.^1,8,9^ Detailed description of variable operational definition is available in Table S1 of Supplementary file 1.

###  Statistical Analysis

 Technical efficiency reflects the extent to which the current state of technology can produce the maximum outcome attainable at each level of the explanatory variables.^26-29^ Although many studies have investigated the efficiency of nursing home care,^30,31^ little is known about the technical efficiency of community-based prevention services for older people.

 Following the conventional model used in technical efficiency estimation, we used the Cobb–Douglas production function, which represents the associations between explanatory variables and outcomes.^26-27,32-34^ In this study, the outcome (transformed O/E ratio) is assumed to be produced by the explanatory variables (the quantities of preventive benefits, including adult day care, home care, and other nursing care; and the quantities of community programs, including functional screening, functional training, health education, and support for social activities), taking into account covariate factors that affect the population risk of functional dependency (the number of general hospitals and clinics, social welfare costs for older people, the proportion of single households, prefecture-level numbers of home- and community-based long-term care providers, financial capacity index, population density, and a year dummy).

 Because the outcome can be affected by unobserved potential confounders that vary across local regions and is unlikely to change over the studied period (eg, local culture and social norms regarding the family provision of informal long-term care and the substitutional use of formal long-term care), we used a fixed effects model, which can account for unobserved time-invariant confounders. We used stochastic frontier analysis with a true fixed effects model (TFEM) for the estimations.^26-29^ We assumed a 1-year time lag because changes in functional dependency status may occur within a year after preventive interventions.^35-38^ Technical efficiency ranges from 0 (lowest) to 1 (highest), and a higher value indicates higher technical efficiency.The parameters were estimated using the maximum likelihood method. We used the “sfpanel” command with the “model (tfe)” option in Stata 16.0 (StataCorp, College Station, TX, USA).^39,40^ The technical details of the analytic methods are provided in Supplementary file 2. In the estimated production function, the coefficients of explanatory variables represent the magnitude of “associations between prevention services and functional dependency” and unexplained variations excluding random error terms represent the level of “technical efficiency.” We used the production function rather than the cost function because public LTCI insurers are expected to maximize the functional independency of older people with limited resources, rather than minimizing costs with a fixed amount of services without considering effects on functional status.

 As a robustness check, we ran six different models. First, we estimated technical efficiency using different distributions of inefficiency terms^28,29,39^ to check the robustness of the stochastic frontier analysis inefficiency assumptions. Second, a translog model was estimated as a flexible functional form, which added all combinations of squared terms and interactions between explanatory variables. Third, to check robustness across the time period, models with no time lag and a 2-year time lag were run. Fourth, to remove the effect of minor changes in regional policy, 17 insurers that expanded the community program target population after 2012 were excluded from the data.^10^ Fifth, outlying values for the outcome, explanatory variables, and covariate factors (ie, values higher than the 75th percentile + 1.5 interquartile and values lower than the 25th percentile − 1.5 interquartile) were excluded from the analysis. Finally, TFEM was conducted using only balanced data (433 insurers over 6 years) to check the effect of unbalanced data.

## Results

 The descriptive statistics are shown in Table 1. From 2009 to 2014, the population risk of moderate functional dependency slightly increased: the median transformed O/E ratio was 0.87 in 2009, 0.67 in 2012, and 0.76 in 2014 (Figure S2 in Supplementary file 1). Of preventive benefits, adult day care was the most popular compared with home care and other nursing care. Regarding community programs, LTCI insurers provided functional screening to an average of 35% of people aged ≥65 years and health education to 30%, but the provision of functional training and support for social activities was limited. The service disparities among insurers, as measured by the coefficient of variance, were smaller for preventive benefits than for community programs.

**Table 1:** Descriptive Statistics of Outcome, Explanatory Variables, and Covariate Factors

**Variable**	**Measurement**	**Mean**	**SD**	**Minimum**	**Maximum**	**CV**
**Outcome**						
Population risk of moderate functional dependency	Transformed sex- and age-adjusted ratio of observed to expected number of individuals aged ≥65 years certified for care required levels 1–2	0.78	0.15	0.01	1.40	0.20
**Explanatory Variables**^a^						
Preventive benefits						
Home care	Number of benefit units^b^ per person aged ≥65 years certified for assistance required levels 1–2	7251	2074	1176	15 347	0.29
Adult day care		16 644	4954	5836	36 905	0.30
Other nursing care		6861	1862	2811	25 312	0.27
Community programs						
Functional screening	Proportion of people aged ≥65 years who received functional screening	0.35	0.21	0.00	0.92	0.61
Functional training	Proportion of people aged ≥65 years who received functional training	0.01	0.01	0.00	0.09	0.99
Health education	Proportion of people aged ≥65 years who received health education	0.30	0.42	0.00	7.15	1.43
Support for social activities	Number of supports for social activities conducted per person aged ≥65 years	0.02	0.06	0.00	0.68	2.83
**Covariate Factors**						
Hospitals and clinics	Number of general hospitals and clinics per 100 000 population	82	27	34	382	0.33
Social welfare costs	Social welfare costs per person aged ≥65 years (yen)	17 372	10 013	2978	104 285	0.58
Single households	Proportion of single households to total households with persons aged ≥65 years	0.24	0.06	0.10	0.47	0.27
Home- and community-based long-term care providers	Prefecture-level number of home- and community-based long-term care providers per 100 000 population aged ≥65 years	473	83	328	717	0.18
Financial capacity index	Ratio of standard fiscal revenue to standard fiscal demand	0.73	0.23	0.21	1.92	0.31
Population density	Number of people per 1 km^2^	2698	3943	15	22 380	1.46

Abbreviations: SD, standard deviation; CV, coefficient of variance (the standard deviation of the value divided by the mean). The data are from 474 insurers in 2009–2014 (n = 2799). Table S1 in Supplementary file 1 provides measurement details. ^a^Preventive benefits exclusively target individuals with assistance required levels 1–2. Because target populations of community programs vary by LTCI insurers and programs, the quantities of each program are calculated using the population aged ≥65 years as the target. ^b^Benefit units are set to reflect the volume of each type of benefit and are linked to the average cost of benefits; one unit is approximately 10 yen and is adjusted by region.

 The TFEM for estimating technical efficiency is shown in Table 2. The coefficients for all explanatory variables except functional screening were positive and statistically significant, suggesting that these services reduce the population risk of moderate functional dependency. Preventive benefits had a larger marginal increase in outcome (ie, transformed O/E ratio) than community programs. Specifically, for preventive benefits, the mean changes in outcome per 1% increase in adult day care, other nursing care, and home care were 0.13%, 0.07%, and 0.04%, respectively. For community programs, the mean changes in outcome per 1% increase in functional training, health education, and support for social activities were 0.03%, 0.01%, and 0.01%, respectively. For covariate factors, the financial capacity index was 0.13%, as expected, suggesting that greater financial resources reduce the population risk of moderate functional dependency. The mean changes in outcome by year compared with 2009 were negative, possibly indicating an increase in care demand and LTCI application in response to a policy that restricted preventive benefits coverage from 2015. Contrary to our expectation, a negative mean change in outcome was found for social welfare costs, and the other covariate factors showed null results.

**Table 2:** Estimated Production Function Using Stochastic Frontier Analysis With a True Fixed Effects Model for 474 Insurers With Population of 50 000 to <2 000 000

**Outcome: ln Population Risk of Moderate Functional Dependency**	**Coefficient**	**95% Confidence Intervals**	***P*** ** Value**
**Explanatory Variables**			
Preventive benefits			
ln Home care	0.04	(0.02, 0.07)	<.001
ln Adult day care	0.13	(0.07, 0.18)	<.001
ln Other nursing care	0.07	(0.05, 0.10)	<.001
Community programs			
ln Functional screening	−0.0001	(−0.003, 0.003)	0.95
ln Functional training	0.03	(0.03, 0.03)	<.001
ln Health education	0.01	(0.01, 0.02)	<.001
ln Support for social activities	0.01	(0.01, 0.01)	<.001
**Covariate Factors**			
ln Hospitals and clinics	0.04	(−0.11, 0.19)	.59
ln Social welfare costs	−0.03	(−0.05, −0.02)	<.001
ln Single households	0.02	(−0.07, 0.11)	.69
ln Home- and community-based long-term care providers	0.04	(−0.05, 0.12)	.38
ln Financial capacity index	0.13	(0.02, 0.23)	.02
ln Population density	0.16	(−0.13, 0.44)	.28
Year (ref: 2009)			
2010	−0.12	(−0.13, −0.11)	<.001
2011	−0.21	(−0.22, −0.19)	<.001
2012	−0.01	(−0.04, 0.01)	.31
2013	−0.08	(−0.10, −0.06)	<.001
sigma_u (standard deviation of inefficiency term)	0.13	(0.13, 0.14)	<.001
sigma_v (standard deviation of random error term)	0.00001	(0.00, 0.00)	.17
Log likelihood	2967.56		
Wald chi-square statistic	650 138.68		<.001

The data are from 474 insurers in 2009–2014 assuming a 1-year time lag (n = 2285). The outcome is the transformed sex- and age-adjusted ratio of the observed to expected number of individuals aged ≥65 years certified for care required levels 1–2; higher outcome values indicate a lower population risk of moderate functional dependency. All variables except for a year dummy were naturally log transformed.

 The median technical efficiency was 0.94 and remained high from 2009 (0.92) to 2013 (0.94) (Figure in the text and Table S2 in Supplementary file 3). The efficiency gaps among LTCI insurers remained almost constant, with an interquartile range of 0.89–0.99. Considering that technical efficiency ranges from 0 (lowest) to 1 (highest), these findings indicate that LTCI insurers in Japan operated prevention services highly efficiently across regions. Low or moderate correlations were found between estimated technical efficiency and the fixed effect terms by year, and ranged from −0.45 to 0.54, suggesting separation of the two estimates.

**Figure:**
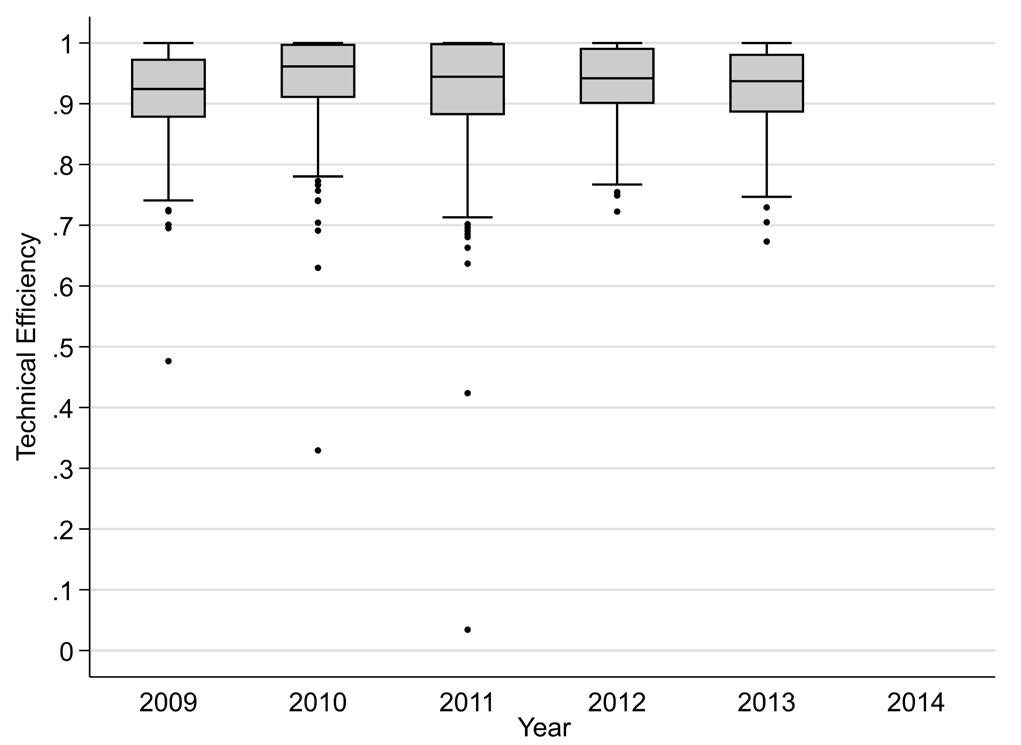
Technical Efficiency of Prevention Services of 474 Insurers With Population of 50 000 to <2 000 000. Efficiency estimation was based on stochastic frontier analysis with a TFEM as shown in Table 2. The median, interquartile range, upper and lower adjacent values (the most extreme values within the 75th percentile + 1.5 interquartile and the 25th percentile − 1.5 interquartile, respectively), and outlying values are indicated by lines, boxes, whiskers, and dots, respectively.

 The production function coefficients were similar to those of the main results for all models used as robustness checks. However, except for the model that excluded outlying values, the standard deviation of the inefficiency term (ie, sigma_u), and/or the standard error of fixed effects, and the standard error of interaction terms of the explanatory variables did not converge, suggesting that remaining heteroscedasticity in the dataset may have biased the estimates.

## Discussion

 In Japan, prevention for functional dependency has been integrated into public LTCI provision. Using a novel application of efficiency measurement with the use of national data from LTCI insurers, we demonstrated that LTCI insurers operate highly efficient prevention services across regions. These findings could contribute to current discussions about the range of benefit coverage in public LTCI systems by presenting a useful option for preventive benefits.

 Previous studies have shown limited and inconsistent evidence for the effectiveness of prevention for functional dependency, except for physical activity and exercise.^5,12,35-38,41^ However, the present findings indicate that all types of services (except functional screening) had marginal gains in outcome. We found that preventive benefits, especially adult day care, had larger marginal gains than community programs. Although we cannot specify the mechanisms by which each service affects older people’s behavior, standardized and targeted nursing care and daily living support may have larger associations with functional dependency than community programs that allow municipality discretion to meet local needs. The limited marginal gains of functional screening may be due to low response rates and a lack of standardized methods of follow-up for people with mild care needs.

 We found that the technical efficiency of prevention services remained high (median: 0.94) across regions (interquartile range: 0.89–0.99). The results demonstrate that nationwide efficient prevention measures are possible within the public LTCI system, through decentralized operation by local insurers under standardized quality control by the central government for insurance reimbursement purposes.^5,9^ This operational scheme in Japan could be applied to LTCI systems in other countries in which each local government operates and finances long-term care under national initiatives.

 This study provides empirical policy evidence to inform the optimal allocation of resources among people with mild care needs, an issue that is currently neglected in debates about LTCI.^2-4^ Given that the average expenditures on preventive benefits and community programs accounted for only approximately 5% and 0.5%, respectively, of the total LTCI expenditures in Japan in 2014,^8^ preventive services are feasible options for individuals who need mild care. Although early stage LTCI systems often limit benefits to those with the highest care needs,^4^ the introduction of preventive services is a feasible option for rapidly aging populations like that of Japan. The introduction of local prevention services into public LTCI systems requires the sustainable design of operational units, regional service delivery, and centralized quality control, in addition to financing. However, in Japan, some preventive benefits were no longer covered by LTCI benefits since 2015.^8,9^ This was an attempt by the central government to contain LTCI costs; though, the cost saving from this measure was probably marginal^1^ and this policy change was made with no evaluation of preventive services. Optimal resource allocations in public LTCI require a systematic and ongoing evaluation system to assess effectiveness and efficiency of benefits.

 This study had several limitations. First, Japan’s LTCI does not screen all people with mild care needs in local regions so the outcome may have been partly affected by the LTCI application process, although we adjusted care demand and supply variables as covariates. A government report estimated that approximately 80% of frail people aged ≥65 years use formal LTCI care.^5^ Second, there were other potential covariate factors. Although the proportion of single households was included, caregiver characteristics that affect care demand, such as gender and marital status,^42^ were not included owing to the lack of data. The prevalence of disease related to demand for long-term care (eg, Alzheimer’s disease and stroke) was not included owing to a lack of LTCI insurer-level data. We did not use variables to surrogate the capacity of older people because regional education level data were only available for the year 2010,^19^ and were completely absorbed into the fixed effect terms in the TFEM. The LTCI premium category as a measure of household income^8^ was removed to avoid high multicollinearity. Third, private sector prevention programs were not included owing to a lack of data. However, individuals with a stronger preference for functional independency are more likely to purchase private services with full out-of-pocket payment. In contrast, the public prevention services included in this study were offered and open to all community-dwelling older people certified as eligible by local public insurers following the nationwide protocol; thus, selection bias from the selective purchase of private services was unlikely. Fourth, efficiency analyses cannot address causality, which is a typical weakness of the established efficiency econometric models used in this study.^26-33^ Efficiency estimation biases in the presence of endogeneity have been noted previously.^43^ We used a fixed effects model to account for unobserved time-invariant heterogeneity, although our estimation may be susceptible to unobserved time-variant heterogeneity. Instrumental variable methods can be used in models where the explanatory variables correlate with the error term. However, we were unable to identify appropriate instrumental variables in this study. Thus, we cannot rule out the possibility of reverse causality. We still believe that at least theoretically reverse causality is unlikely in our data because the estimated coefficients for all prevention services (except for functional screening) shown in Table 2 were all in the theoretically expected direction, supporting their preventive associations. Nevertheless, the association may be confounded by the baseline outcomes. The causal relationship between prevention services and functional dependency needs to be further examined in future studies. Fifth, differences in production function by population size remain unknown because we analyzed insurers with a specific population size to remove random variations and maintain sample homogeneity. In a supplemental analysis using insurers with populations of 10 000 to <50 000, smaller but positive coefficients for the explanatory variables were found (Table S3 and Figure S3 in Supplementary file 3). This suggests that scale economy of production function should be investigated in future studies. Finally, the cost-effectiveness of prevention services was not investigated owing to the limited available information on quality of life in functionally dependent older people and future cost savings with a suitable time horizon. Additional studies are needed to address these issues.

## Conclusion

 This study demonstrated that public LTCI insurers in Japan operate highly efficient prevention services across regions. In addition to covering older people who already need long-term care, the introduction of prevention services is worth considering to reduce the population risk of moderate functional dependency. Decentralized operation and standardized quality control could potentially achieve equivalent service efficiency across regions.

## Acknowledgements

 We would like to express our gratitude to municipal LTCI staff members for informing us about local prevention services and how they were reported in national surveys.

## Ethical issues

 Because the study involved secondary analysis of anonymous data, the requirements for ethical approval and informed consent were waived under governmental-use approval.

## Competing interests

 Authors declare that they have no competing interests.

## Disclaimer

 The views expressed in the article are the authors’ own and not an official position of the affiliated institutions.

## Supplementary file


Supplementary file 1. Flowchart of Sample Selection and Detailed Description of Variable Measurement.

Supplementary file 2. The Details of Stochastic Frontier Analysis With a True Fixed Effects Model.

Supplementary file 3. Estimated Production Function and Technical Efficiency Score.
