# Supplementary material for: Technical Efficiency of Prevention Services for Functional Dependency in Japan’s Public Long-term Care Insurance System: An Ecological Study
Source: Int J Health Policy Manag. 2024 Jun 19;13:8226. doi: 10.34172/ijhpm.8226 (PMC11365088; doi:10.34172/ijhpm.8226)
Supplement: Supplementary file 1 — Flowchart of Sample Selection and Detailed Description of Variable Measurement. [file ijhpm-13-8226-s001.pdf]

Article title: Technical Efficiency of Prevention Services for Functional Dependency in Japan's Public Long-term Care Insurance System: An Ecological Study

Journal name: International Journal of Health Policy and Management (IJHPM)

Authors' information: Ayumi Hashimoto[1]*, Hideki Hashimoto[2], Hiroyuki Kawaguchi[3]

[1]Graduate School of Medicine, The University of Tokyo, Tokyo, Japan

[2]Department of Health and Social Behavior, School of Public Health, The University of Tokyo, Tokyo, Japan

[3]Economics Faculty, Seijo University, Tokyo, Japan

***Correspondence to:** Ayumi Hashimoto; Email: ahashimoto140@gmail.com

Citation: Hashimoto A, Hashimoto H, Kawaguchi H. Technical efficiency of prevention services for functional dependency in Japan's public long-term care insurance system: an ecological study. Int J Health Policy Manag. 2024;13:8226. doi:10.34172/ijhpm.8226

Supplementary file 1. Flowchart of Sample Selection and Detailed Description of Variable Measurement

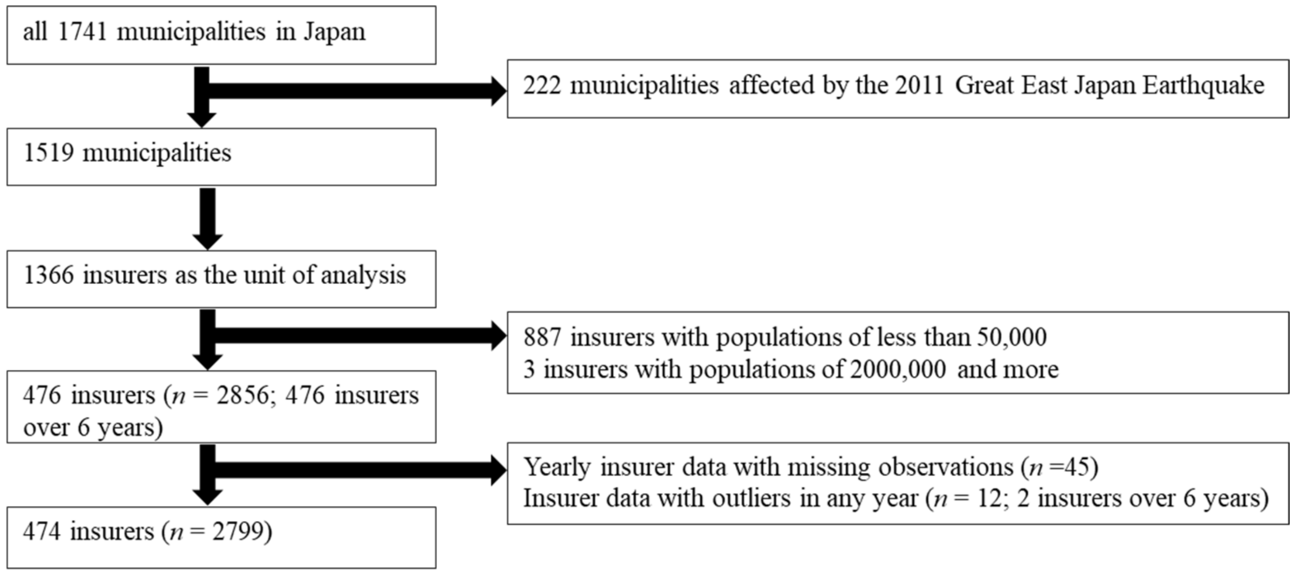

Figure S1. Flowchart of the study sample selection of 474 insurers from 1741 municipalities

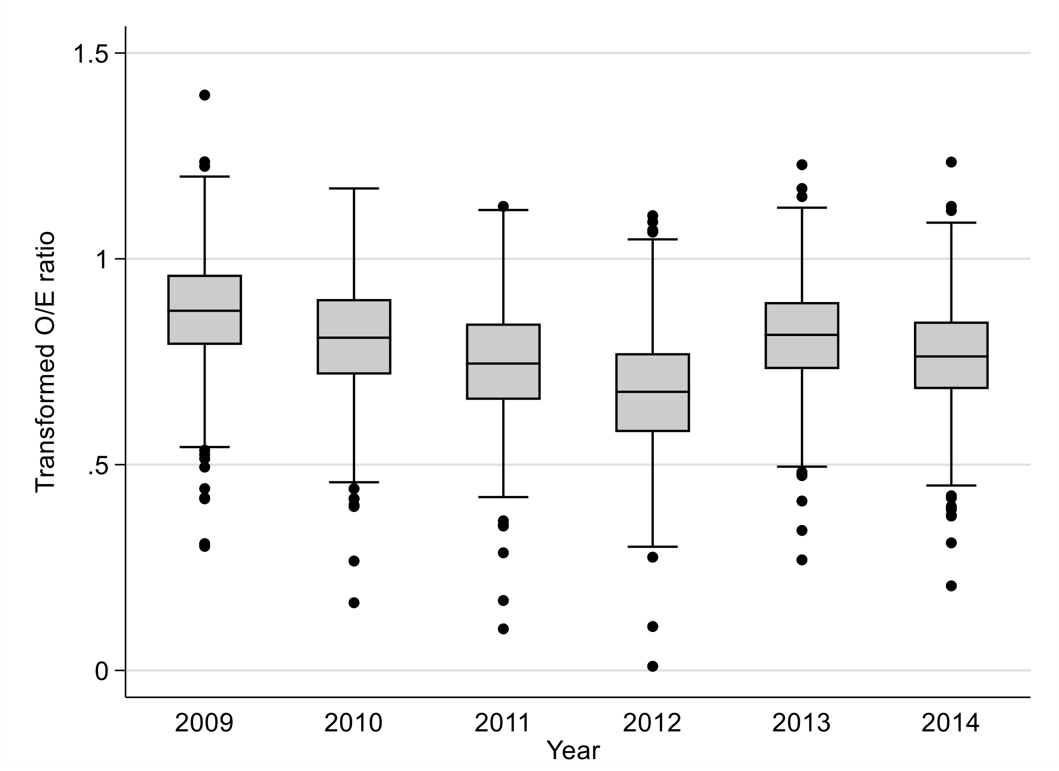

Figure S2. Time trend of population risk of moderate functional dependency of 474 insurers

The transformed O/E ratio is the transformed sex- and age-adjusted ratio of the observed to expected number of individuals aged ≥65 years certified for care required levels 1–2; higher outcome values indicate a lower population risk of moderate functional dependency. The median, interquartile range, upper and lower adjacent values (the most extreme values within the 75th percentile + 1.5 interquartile and the 25th percentile − 1.5 interquartile, respectively), and outlying values are indicated by lines, boxes, whiskers, and dots, respectively.

Table S1. Details of measurement of outcome, explanatory variables, and covariate factors

Variable	Measurement details	Source	Surrogate and reasons
**Outcome**			
Population risk of moderate functional dependency	Transformed sex- and age-adjusted ratio of observed to expected (O/E) number of individuals aged ≥65 years certified for care required levels 1–2:<br><br>To obtain the sex- and age-adjusted O/E ratio, the expected number was calculated by multiplying the national average certification rate by sex (men, women) and 5-year age group (65–69, 70–74, 75–79, 80–84, 85–89, ≥90 years) in 2014 for care required level 1 and care required level 2 by the corresponding population number by sex and 5-year age group for each insurer, and summing them. The sex- and age-adjusted O/E ratio is the actual observed number of individuals for CL 1–2 divided by the obtained expected number of individuals for CL 1–2. The population data for 2010 and 2015 were allocated to 2009–2012 and 2013–2014, respectively<br><br>When calculating the transformed O/E ratio, we subtracted the O/E ratio from the median O/E ratio to reverse the positive and negative values for ease of interpretation; zero values were replaced with 0.01 for log transformation using the Cobb–Douglas functional form	Survey of long-term care insurance services 2009–2014; population census 2010, 2015	Higher outcome values indicate lower population risk of moderate functional dependency because care required levels 1–2 represent moderate care needs
**Explanatory variables**			
Preventive benefits (home care, adult day care, and other nursing care)	Number of benefit units per person aged ≥65 years certified for assistance required levels 1–2: Limited to people aged ≥65 years, the total number of benefit units for the fiscal year divided by the number of people certified for assistance required levels 1–2 at the end of the fiscal year	Survey of long-term care insurance services 2009–2014	Values indicate quantities of each prevention service

Functional screening	Proportion of people aged ≥65 years who received functional screening:<br>The cumulative total number of implementers/respondents was used for 2009–2013, and the actual number of implementers was used in 2014	Survey of long-term care prevention and daily life support programs 2009–2014; the value of “1–3” was replaced by 2 in 2009, and “0–3” was replaced by 1.5 in 2010	
Functional training	Proportion of people aged ≥65 years who received functional training:<br>The actual number of people who received functional training was used		
Health education	Proportion of people aged ≥65 years who received health education:<br>The cumulative total number of people who received health education was used		
Support for social activities	Number of supports for social activities conducted per person aged ≥65 years:<br>The cumulative total number of supports for social activities was used		
**Covariate factors**			
Hospitals and clinics	Number of general hospitals and clinics per 100,000 population:<br>The population data for 2010 and 2015 were allocated to 2009–2012 and 2013–2014, respectively	Survey of medical institutions 2009–2014; population census 2010, 2015	Access to medical care is indicated by density of medical care
Social welfare costs	Social welfare costs per person aged ≥65 years (yen):<br>Social welfare costs for older people include personnel, property, maintenance, repair, social assistance, and subsidies. Transfers to long-term care insurance account and medical care for latter-stage elderly account (mainly those aged ≥75 years) were excluded	Local finance statistics annual report 2009–2014	Access to social welfare resources is indicated by average social welfare cost per older person
Single households	Proportion of single households to total households with persons aged ≥65 years:<br>The data for 2010 and 2015 were allocated to 2009–2012 and 2013–2014, respectively	Population census 2010, 2015	Informal care capacity, which leads to long-term care demand, is

			indicated by proportion of single households
Home- and community-based long-term care providers	Prefecture-level number of home- and community-based long-term care providers per 100,000 population aged ≥65 years:<br>The sum of the number of home- and community-based long-term care providers for each service was used. The number of all facilities in action was reported in the comprehensive online survey from 2012 to 2014; the number of facilities in action that responded to the mail survey was reported from 2009 to 2011. Therefore, for 2009–2011, the number reported was multiplied by the inverse of the survey response rate	Survey of institutions and establishments for long-term care 2009–2014	Long-term care supply is indicated by density of home- and community-based long-term care providers
Financial capacity index	Ratio of standard fiscal revenue to standard fiscal demand:<br>The index was averaged for the last 3 years. If municipalities merged, incorporated, or jointly ran long-term care insurance, the index was weighted by the population size of each municipality in 2010	Local finance statistics annual report 2009–2014	The abundance and convenience of outdoor spaces and transportation are indicated by financial resources and population density
Population density	Number of people per 1 km^2:<br>The data for 2010 and 2015 were allocated to 2009–2012 and 2013–2014, respectively	Population census 2010, 2015	
A year dummy	Year dummy from 2009 to 2013 because we used the model with a 1-year time lag.		Changes in care demand and supply in response to a policy that restricted preventive benefits coverage from 2015 is indicated by year dummy
