# Supplementary material for: Technical Efficiency of Prevention Services for Functional Dependency in Japan’s Public Long-term Care Insurance System: An Ecological Study
Source: Int J Health Policy Manag. 2024 Jun 19;13:8226. doi: 10.34172/ijhpm.8226 (PMC11365088; doi:10.34172/ijhpm.8226)
Supplement: Supplementary file 2 — The Details of Stochastic Frontier Analysis With a True Fixed Effects Model. [file ijhpm-13-8226-s002.pdf]

Article title: Technical Efficiency of Prevention Services for Functional Dependency in Japan's Public Long-term Care Insurance System: An Ecological Study

Journal name: International Journal of Health Policy and Management (IJHPM)

Authors' information: Ayumi Hashimoto[1]*, Hideki Hashimoto[2], Hiroyuki Kawaguchi[3]

[1]Graduate School of Medicine, The University of Tokyo, Tokyo, Japan

[2]Department of Health and Social Behavior, School of Public Health, The University of Tokyo, Tokyo, Japan

[3]Economics Faculty, Seijo University, Tokyo, Japan

***Correspondence to:** Ayumi Hashimoto; Email: ahashimoto140@gmail.com

Citation: Hashimoto A, Hashimoto H, Kawaguchi H. Technical efficiency of prevention services for functional dependency in Japan's public long-term care insurance system: an ecological study. Int J Health Policy Manag. 2024;13:8226. doi:10.34172/ijhpm.8226

Supplementary file 2. The Details of Stochastic Frontier Analysis With a True Fixed Effects Model

For technical efficiency estimation, we used stochastic frontier analysis because it accounts for random error and is more robust to variable changes and outliers than nonparametric data envelopment analysis.[26-29] However, technical efficiency estimation with stochastic frontier analysis is confounded by sample heterogeneity. Greene proposed the true fixed effects model (TFEM),[28,29] which can distinguish unobserved time-invariant heterogeneity from estimated technical efficiency. The TFEM assumes that technical efficiency varies over time without any time function, and therefore should better reflect the actual technical efficiency of insurers. Based on the results of the F-test in the pooled ordinary least squares model (P < 0.001) and the Hausman test (P < 0.001), a TFEM was selected to estimate technical efficiency in this study.

The conventional Cobb–Douglas production form was used, as shown in equation (1):

$$\ln Y_{it+1} = \alpha_i + \beta_1 \ln X_{it} + \beta_2 \ln Z_{it} + v_{it} - u_{it}, \qquad (1)$$

$$v_{it} \sim N\left[0, \sigma^2_v\right],\ u_{it} = \left|U_{it}\right|,\ U_{it} \sim N\left[0, \sigma^2_u\right].$$

Y, X, and Z are outcome, explanatory variables, and covariate factors, respectively. Long-term care insurance (LTCI) insurer is denoted by i, year is denoted by t, and the random error term is denoted by v_{it}. u_{it} is the inefficiency term, which is assumed to be half-normally distributed. α_i is the time-invariant insurer-specific fixed effect (heterogeneity), which may represent unobserved potential confounders that vary across local regions and is unlikely to change over the studied period (e.g., local culture and social norms regarding the family provision of informal long-term care and the substitutional use of formal long-term care). Y, X, and Z were naturally log-transformed. We assumed a 1-year time lag because changes in functional dependency status may occur within a year after preventive interventions.[35-38] The parameters were estimated using the maximum likelihood method. The efficiency score was estimated as $\exp[-E(u_{it} \mid v_{it} - u_{it})]$ based on the estimator proposed by Jondrow et al.[39,40] We used the “sfpanel” command with the “model (tfe)” option in Stata 16.0 (StataCorp, College Station, TX, USA).

In health economics, production function is classified into two categories: production function of *health care* and production function of *health*. The former produces health care services with inputs of labor and capital, and the latter produces health with inputs of health care services.[32-34] In this study, we used the production function of *health* because public LTCI insurers are expected to maximize the functional independency of older people with a limited amount of preventive services.

References for Supplementary file 2

26. Coelli TJ, Rao DSP, O'Donnell CJ, Battese GE. An introduction to efficiency and productivity analysis. New York: Springer; 2005. doi:10.1007/b136381
27. Farrell MJ. The measurement of productive efficiency. *Journal of the Royal Statistical Society: Series A.* 1957;120(3):253-290. doi:10.2307/2343100
28. Greene W. Distinguishing between heterogeneity and inefficiency: Stochastic frontier analysis of the World Health Organization's panel data on national health care systems. *Health Econ.* 2004;13(10):959-980. doi:10.1002/hec.938
29. Greene W. Reconsidering heterogeneity in panel data estimators of the stochastic frontier model. *J Econom*. 2005;126(2):269-303. doi:10.1016/j.jeconom.2004.05.003
32. Cohen JP. Production functions for medical services. In: Culyer AJ, eds. *Encyclopedia of health economics, Volume 3.* Amsterdam: Elsevier; 2014:180-183.
33. Hollingsworth B. Evaluating efficiency of a health care systemin the developed world. In: Culyer AJ, eds. *Encyclopedia of health economics, Volume 1.* Amsterdam: Elsevier; 2014:292-299.
34. Hashimoto A, Kawaguchi H, Hashimoto H. Contribution of the technical efficiency of public health programs to national trends and regional disparities in unintentional childhood injury in Japan. *Front Public Health.* 2022;10:913875. doi: 10.3389/fpubh.2022.913875.
35. Lunt C, Dowrick C, Lloyd-Williams M. What is the impact of day care on older people with long-term conditions: A systematic review. *Health Soc Care Community.* 2021;29(5):1201-1221.

doi:10.1111/hsc.13245

36. Negm AM, Kennedy CC, Thabane L, et al. Management of frailty: A systematic review and network meta-analysis of randomized controlled trials. *J Am Med Dir Assoc.* 2019;20(10):1190-1198. doi:10.1016/j.jamda.2019.08.009
37. Puts MTE, Tobasi S, Andrew MK, et al. Interventions to prevent or reduce the level of frailty in community-dwelling older adults: A scoping review of the literature and international policies. *Age Ageing.* 2017;46(3):383-392. doi:10.1093/ageing/afw247
38. Boland L, Légaré F, Perez MM, et al. Impact of home care versus alternative locations of care on elder health outcomes: an overview of systematic reviews. *BMC Geriatr.* 2017;17(1):20. doi:10.1186/s12877-016-0395-y
39. Belotti F, Daidone S, Ilardi G, Atella V. Stochastic frontier analysis using Stata. *The Stata Journal.* 2013;13:719-58. doi:10.1177/1536867X1301300404
40. Jondrow J, Knox Lovell CA, Materov IS, Schmidt P. On the estimation of technical inefficiency in the stochastic frontier production function model. *J Econom.* 1982;19:233-238. doi: 10.1016/0304-4076(82)90004-5
