# Supplementary material for: Technical Efficiency of Prevention Services for Functional Dependency in Japan’s Public Long-term Care Insurance System: An Ecological Study
Source: Int J Health Policy Manag. 2024 Jun 19;13:8226. doi: 10.34172/ijhpm.8226 (PMC11365088; doi:10.34172/ijhpm.8226)
Supplement: Supplementary file 3 — Estimated Production Function and Technical Efficiency Score. [file ijhpm-13-8226-s003.pdf]

Article title: Technical Efficiency of Prevention Services for Functional Dependency in Japan's Public Long-term Care Insurance System: An Ecological Study

Journal name: International Journal of Health Policy and Management (IJHPM)

Authors' information: Ayumi Hashimoto[1]*, Hideki Hashimoto[2], Hiroyuki Kawaguchi[3]

[1]Graduate School of Medicine, The University of Tokyo, Tokyo, Japan

[2]Department of Health and Social Behavior, School of Public Health, The University of Tokyo, Tokyo, Japan

[3]Economics Faculty, Seijo University, Tokyo, Japan

***Correspondence to:** Ayumi Hashimoto; Email: ahashimoto140@gmail.com

Citation: Hashimoto A, Hashimoto H, Kawaguchi H. Technical efficiency of prevention services for functional dependency in Japan's public long-term care insurance system: an ecological study. Int J Health Policy Manag. 2024;13:8226. doi:10.34172/ijhpm.8226

Supplementary file 3. Estimated Production Function and Technical Efficiency Score

Table S2. Estimated technical efficiency score for 474 insurers with population of 50 000 to <2 000 000

Year	N	Median	Interquartile range
2009	474	0.92	(0.88, 0.97)
2010	447	0.96	(0.91, 1.00)
2011	436	0.94	(0.88, 1.00)
2012	460	0.94	(0.90, 0.99)
2013	468	0.94	(0.89, 0.98)
2014	-	-	-
2009–13	2285	0.94	(0.89, 0.99)

Efficiency estimation was based on a true fixed effects model as shown in Table 2.

Table S3. Estimated production function using stochastic frontier analysis with a true fixed effects model for insurers with population of 10 000 to <50 000

**Outcome**: ln Population risk of moderate functional dependency	**Coefficient**	**95% confidence intervals**	***P*-value**
**Explanatory variables**			
Preventive benefits			
ln Home care	−0.03	(−0.04, −0.02)	<0.001
ln Adult day care	0.06	(0.05, 0.07)	<0.001
ln Other nursing care	0.06	(0.05, 0.07)	<0.001
Community programs			
ln Functional screening	0.0002	(−0.001, 0.001)	0.67
ln Functional training	0.005	(0.002, 0.01)	0.001
ln Health education	−0.001	(−0.001, −0.001)	<0.001
ln Support for social activities	0.001	(0.001, 0.002)	0.001
**Covariate factors**			
ln Hospitals and clinics	−0.01	(−0.04, 0.01)	0.30
ln Social welfare costs	−0.004	(−0.01, −0.001)	0.001
ln Single households	−0.04	(−0.11, 0.04)	0.33
ln Home and community-based long-term care providers	0.06	(−0.03, 0.14)	0.17
ln Financial capacity index	0.02	(−0.05, 0.09)	0.61
ln Population density	0.43	(0.36, 0.51)	<0.001
Year (ref: 2009)			
2010	−0.09	(−0.10, −0.09)	<0.001
2011	−0.17	(−0.17, −0.16)	<0.001
2012	−0.06	(−0.07, −0.05)	<0.001
2013	−0.07	(−0.10, −0.05)	<0.001
sigma_u (standard deviation of inefficiency term)	0.14	(0.14, 0.14)	<0.001
sigma_v (standard deviation of random error term)	0.00001	(0.00, 0.00)	0.38
Log likelihood	3132.51		
Wald chi-square statistic	4480000		<0.001

The data are from 520 insurers in 2009–2014 assuming a 1-year time lag (n = 2533). The outcome is transformed sex- and age-adjusted ratio of the observed to expected number of individuals aged ≥65 years certified for care required levels 1–2; higher outcome values indicate a lower population risk of moderate functional dependency. All variables except for a year dummy were naturally log-transformed.

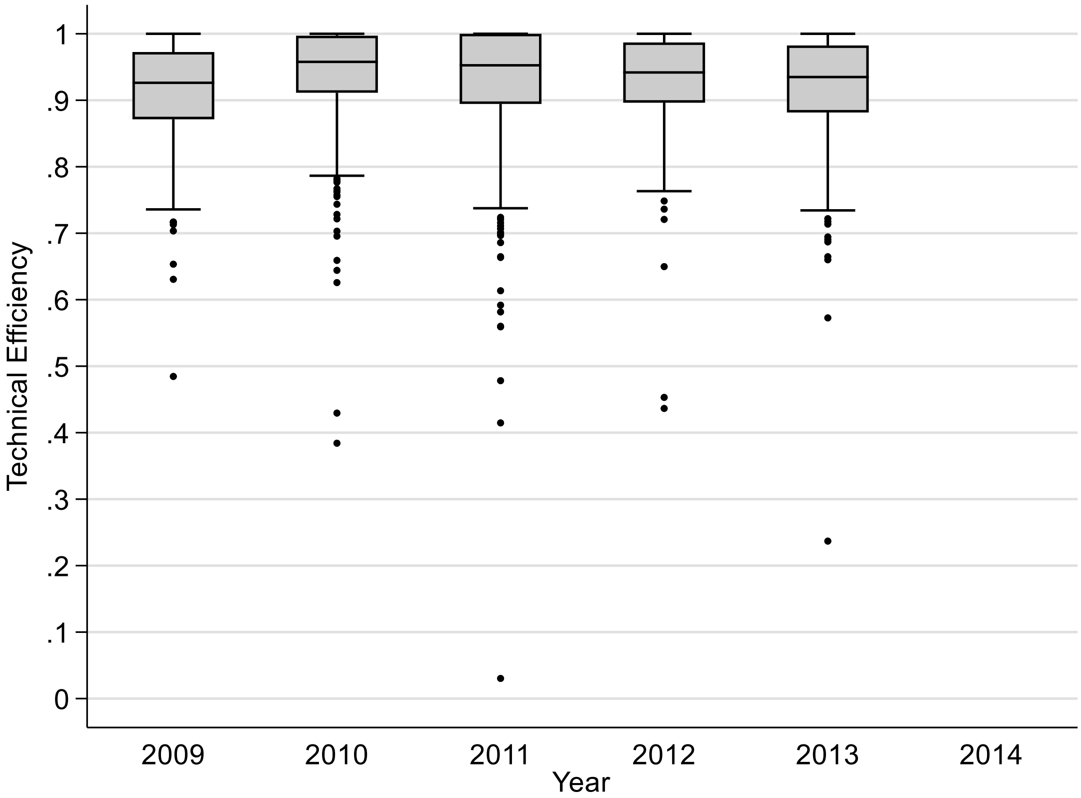

Figure S3. Technical efficiency of prevention programs of 520 insurers with population of 10 000 to <50 000

Efficiency estimation was based on stochastic frontier analysis with a true fixed effects model as shown in Table S3. The median, interquartile range, upper and lower adjacent values (the most extreme values within the 75th percentile + 1.5 interquartile and the 25th percentile − 1.5 interquartile, respectively), and outlying values are indicated by lines, boxes, whiskers, and dots, respectively.
